# Violence: heightened brain attentional network response is selectively muted in Down syndrome

**DOI:** 10.1186/s11689-015-9112-y

**Published:** 2015-06-03

**Authors:** Jeffrey S. Anderson, Scott M. Treiman, Michael A. Ferguson, Jared A. Nielsen, Jamie O. Edgin, Li Dai, Guido Gerig, Julie R. Korenberg

**Affiliations:** Department of Radiology, 1A71 School of Medicine, University of Utah, Salt Lake City, UT 84132 USA; Interdepartmental Program in Neuroscience, University of Utah, Salt Lake City, USA; The Brain Institute at the University of Utah, Salt Lake City, USA; Department of Bioengineering, University of Utah, Salt Lake City, USA; Department of Psychology, University of Arizona, Tucson, USA; Department of Pediatrics, University of Utah, Salt Lake City, USA; Scientific Computing and Imaging Institute, University of Utah, Salt Lake City, USA

**Keywords:** fMRI, Violence, Down syndrome, Attention

## Abstract

**Background:**

The ability to recognize and respond appropriately to threat is critical to survival, and the neural substrates subserving attention to threat may be probed using depictions of media violence. Whether neural responses to potential threat differ in Down syndrome is not known.

**Methods:**

We performed functional MRI scans of 15 adolescent and adult Down syndrome and 14 typically developing individuals, group matched by age and gender, during 50 min of passive cartoon viewing. Brain activation to auditory and visual features, violence, and presence of the protagonist and antagonist were compared across cartoon segments. fMRI signal from the brain’s dorsal attention network was compared to thematic and violent events within the cartoons between Down syndrome and control samples.

**Results:**

We found that in typical development, the brain’s dorsal attention network was most active during violent scenes in the cartoons and that this was significantly and specifically reduced in Down syndrome. When the antagonist was on screen, there was significantly less activation in the left medial temporal lobe of individuals with Down syndrome. As scenes represented greater relative threat, the disparity between attentional brain activation in Down syndrome and control individuals increased. There was a reduction in the temporal autocorrelation of the dorsal attention network, consistent with a shortened attention span in Down syndrome. Individuals with Down syndrome exhibited significantly reduced activation in primary sensory cortices, and such perceptual impairments may constrain their ability to respond to more complex social cues such as violence.

**Conclusions:**

These findings may indicate a relative deficit in emotive perception of violence in Down syndrome, possibly mediated by impaired sensory perception and hypoactivation of medial temporal structures in response to threats, with relative preservation of activity in pro-social brain regions. These findings indicate that specific genetic differences associated with Down syndrome can modulate the brain’s response to violence and other complex emotive ideas.

**Electronic supplementary material:**

The online version of this article (doi:10.1186/s11689-015-9112-y) contains supplementary material, which is available to authorized users.

## Background

Down syndrome is a prevalent intellectual disability syndrome occurring in 9.0 to 11.8 per 10,000 live births [1] associated with intellectual, motor, memory, and language impairments [2]. Despite moderately severe, though variable, cognitive impairments, individuals with Down syndrome are often perceived as particularly affectionate and happy [3]. In contrast to other neurodevelopmental conditions such as autism, where social function is a particular deficit, individuals with Down syndrome, as in Williams syndrome, exhibit some relative strengths in social function compared to other neurocognitive domains [4]. However, aspects of social cognition have shown particular impairments, especially emotion recognition, and in particular, the recognition of surprise and fear [5].

Relatively little is known about brain activation differences in individuals with Down syndrome. Two prior task-based studies have been reported using functional MRI in Down syndrome. During passive story listening, classical language regions showed decreased activation relative to controls in one study [6]. In another study of object recognition, correlations between brain activation and a metric of visuospatial ability were demonstrated in middle and dorsal frontal gyri in Down syndrome but in the occipital and parietal lobes for typically developing individuals [7].

Functional MRI connectivity is also abnormal in Down syndrome. We recently reported that functional connectivity is abnormal in Down syndrome, with generalized increased synchrony between distributed brain networks and deficits in both negatively correlated and long-range positively correlated connections [8]. The finding of between-network hyperconnectivity was also observed independently by Vega and colleagues [9]. Moreover, hyperconnectivity between the anterior temporal and anterior cingulate cortex, in addition to reduced within-network connectivity in the dorsal attention network, was also observed by Pujol and colleagues [10]. These findings contributed to impaired scores on adaptive function testing [10]. In contrast, for a near-infrared spectroscopy study performed on infants with Down syndrome, lower mean connectivity between channels was observed [11].

In our prior report [8], participants watched cartoon audiovisual stimuli for extended periods (50 min per participant) in order to minimize participant motion and maintain wakefulness. Yet the cartoons themselves provide a rich substrate for probing social interactions to protagonists, antagonists, and violence. In particular, the Bugs Bunny cartoons viewed contain extensive social interactions among characters as well as stylized depictions of humorous but extreme cartoon violence such as characters falling off cliffs, characters being assaulted with blunt objects, or characters smoking exploding cigars. These types of scenes receive ubiquitous media attention for potentially adverse consequences on aggressivity and mental health development in children given the large amount of time children spend viewing cartoons, yet we found no characterization in the imaging literature of brain responses to such stylized violence in either typically developing individuals or those with developmental syndromes. We examined functional brain activation in response to such stimuli in Down syndrome and in typical development to determine whether regional brain activation patterns could be characterized, as well as whether atypical neural activation might be present that could provide clues to a brain basis for the deficits seen in Down syndrome.

## Methods

### Participant characteristics

Analyses for this report were performed based on the data from 15 individuals with Down syndrome (mean age 20.2 ± 6.3, range 14–34, 9 males, 6 females) and 14 typically developing participants (mean age 23.7 ± 5.9, range 15–39, 8 males, 6 females), displayed in Table 1. Three participants with Down syndrome and two control participants were left-handed. All study procedures were performed in compliance with guidelines approved by the University of Utah Institutional Review Board. Informed consent or assent (with guardian consent) was obtained from all participants with Down syndrome and controls.

**Table 1:** Demographics of study participants

	Age	VIQ	PIQ
Down syndrome (*n*)	15 (9 M, 6 F)	14	14
DS mean ± s.d.	20.2 ± 6.3 (14–34)	53.1 ± 12.6	44.9 ± 5.4
Control (*n*)	14 (8 M, 6 F)	8	8
Control mean ± s.d.	23.7 ± 5.9 (15–39)	107.6 ± 12.7	111.7 ± 13.8
*p*-Value (two-tailed *t* test)	0.14	5.1 × 10^−9^	5.0 × 10^−13^

All participants with Down syndrome, recruited from the community, underwent genotyping to confirm trisomy 21. One participant exhibited genetic mosaicism for Down syndrome but showed facial, behavioral, and cognitive deficits characteristic of Down syndrome. Group differences in activation were not qualitatively different when this subject was excluded. One additional participant with Down syndrome was excluded from all analyses due to excessive motion during the scan and was not included in the total. Control participants were recruited from the community. For both control and participants with Down syndrome, medical history and structured psychiatric interview (DSM-IV) were performed by an experienced physician in neurodevelopmental disorders [12]. No control participants had a history of developmental, learning, cognitive, neurological, or neuropsychiatric Axis I condition. Verbal IQ (VIQ) and performance IQ (PIQ) were measured with the Kaufman Brief Intelligence Test, Second Edition [13]. IQ measurements were performed in a subset (8/14) of control participants (mean 107.6 ± 12.7 VIQ; 111.7 ± 13.8 PIQ) and 14/15 participants with Down syndrome (53.1 ± 12.6 VIQ; 44.9 ± 5.4 PIQ). One participant with Down syndrome was not able to complete IQ testing. We note that our control cohort exhibits slightly higher IQ than the general population. Six of the participants with Down syndrome were taking no medications at the time of the scan. Other participants were taking levothyroxine (*n* = 5), microgestin (*n* = 1), enalapril (*n* = 1), omeprazole (*n* = 1), lansoprazole (*n* = 1), fluticasone proprionate (*n* = 1), insulin (*n* = 2), albuterol (*n* = 2), and sertraline (*n* = 1).

As a template of the attention control network, comprising both dorsal attention and ventral attention networks, we examined data from 1011 subjects from publicly available datasets released with the open-access 1000 Functional Connectomes Project (http://fcon_1000.projects.nitrc.org) in which resting-state functional magnetic resonance imaging (fMRI) scans have been aggregated from 28 sites [14] as well as typically developing subjects from the ADHD 200 project from the International Neuroimaging Data-sharing Initiative (fcon_1000.projects.nitrc.org/indi/adhd200/index.html) including 8 sites [15]. Data processing and subject selection has been previously described [16]. We calculated mean functional correlation to four 5-mm radius ROIs in the bilateral anterior insula (left anterior insula *x* = −44, *y* = 8, *z* = 4; right anterior insula *x* = 48, *y* = 10, *z* = 0) and intraparietal sulcus (left IPS *x* = −48, *y* = −38, *z* = 51; right IPS *x* = 44, *y* = −38, *z* = 49).

### Image acquisition

Images were acquired on Siemens 3 Tesla Trio scanner with 12-channel head coil. The scanning protocol consisted of initial 1 mm isotropic MPRAGE acquisition for an anatomic Table 1. BOLD echoplanar images (TR = 2.0 s, TE = 28 ms, GRAPPA parallel acquisition with acceleration factor = 2, 40 slices at 3 mm slice thickness, 64 × 64 matrix) were obtained while viewing Bugs Bunny cartoons (Looney Tunes Golden Collection Volume 1, Warner Home Video) [17]. These consisted of 10 5-min clips, none of which contained a complete cartoon. The following 10 clips were used, beginning at the opening credits for each clip: “Baseball Bugs”, “High Diving Hare”, “Bully for Bugs”, “What’s Up Doc”, “Ballot Box Bunny”, “Rabbit of Seville”, “Wabbit Twouble”, “Rabbit’s Kin”, “Long-Haired Hare”, and “Rabbit Seasoning”. Both auditory and visual components of the video were presented. Subjectively, both control and participants with Down syndrome appeared to tolerate the cartoons well and remained awake during the stimuli as observed by live video feed of the participants’ eyes during examination. Data was obtained during 10 five-minute cartoons for each participant, presented in the same order for each participant. The stimulus computer was synchronized to the onset of the first BOLD image via fiber optic pulse emitted by the scanner for reproducible, precise onset timing. Images were presented through LCD projection onto a screen positioned within the bore of the scanner and viewed via a mirror positioned on the top of the head coil. Auditory stimuli were presented through MRI-compatible headphones (Avotec).

### fMRI preprocessing

Offline preprocessing was performed in MATLAB (Mathworks, Natick, MA, USA) using SPM8 (Wellcome Trust, London, UK) software. Initial slice timing correction was performed to adjust for interleaved slice acquisition. All images were motion corrected using the realign procedure. BOLD images were coregistered to the MPRAGE anatomic image sequence for each participant. All images were normalized to the MNI template brain (T1.nii in SPM8), with manual inspection of appropriate normalization in all participants. Spatial smoothing was performed with 8 mm FWHM kernel.

### Statistical analysis

Time courses for all 10 cartoon clips were obtained prior to analysis by 2 observers blinded to study design and data consisting of second-by-second identification of whether (1) the protagonist was on screen (51.3 % of the cartoons), (2) the antagonist was on screen (47.3 % of the cartoons), and (3) a violent act or accident likely to result in bodily harm was taking place against any character (4.2 % of the cartoons). Interrater reliability was assessed using Cohen’s kappa (MATLAB) and found to show κ = 0.65 for protagonist on screen (substantial agreement), 0.55 for villain on screen (moderate agreement), and 0.49 for violent scenes (moderate agreement). Only scenes scored by both observers were used for subsequent analysis.

A general linear model was used to evaluate for brain activation associated with epochs when the protagonist was on screen, antagonist was on screen, and for violent scenes, with multiple regressors including six head motion parameters for each subject, auditory volume, and visual complexity of the video scenes. Auditory volume was assessed by measuring the waveform of the sound presented during the movie and measuring root-mean-square of the signal for timepoints within each brain volume acquired. This time series was convolved with a hemodynamic response function (spm_hrf [2]) and used as a regressor. Visual complexity consisted of the average root-mean-square difference in red, green, and blue pixel intensity from frame to frame, averaged across all pixels and for all frames within each brain volume acquisition. This time series was also convolved with a hemodynamic response function and used as a regressor in the model.

This model generated contrasts in a first level event-related paradigm in each participant, with all 10 cartoons concatenated for each participant. Second-level group analysis consisted of two-tailed *t* tests for each group independently and for DS > control and control > DS, with significant clusters requiring acceptable false discovery rate *q* < 0.05 to account for multiple comparisons.

### Attentional network analysis

To analyze fluctuations in activation in brain attentional regions, we identified a priori a set of voxels comprising the brain’s dorsal attention network from a prior report in the literature [18]. These voxels were used as a restriction mask and average time series for these voxels was calculated for each participant in each cartoon. For each participant and each cartoon, relative activation was obtained by linear detrending, subtracting the mean and dividing by the standard deviation of the BOLD signal. For the mean dorsal attention network activation in each participant sample for each cartoon, 95 % confidence intervals were calculated. Additionally, the temporal autocorrelogram was calculated for each participant’s time series in each cartoon for ±3 lags, with Fisher transformation of the autocorrelograms to improve normality. Average autocorrelograms were obtained for each participant (averaged across all cartoons) and for each cartoon in each participant sample (Down syndrome, control). Averaged measurements were converted back to correlation measurements by an inverse Fisher transformation for display. Full-width half maximum measurements were obtained by performing linear interpolation of datapoints in the averaged autocorrelograms, and a two-tailed *t* test was performed between groups of participants on the FWHM for the autocorrelograms.

## Results

**Fig. 1:**
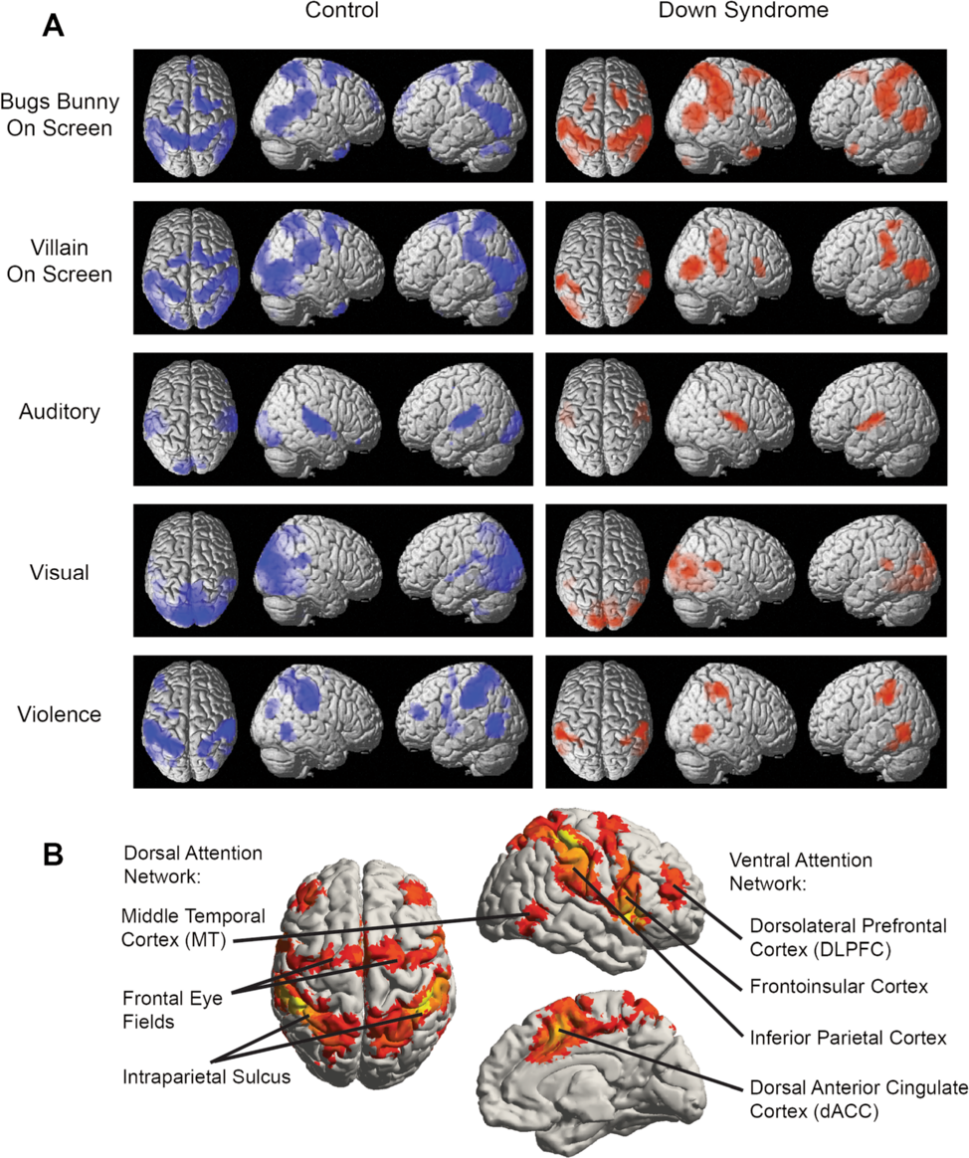
**a** Brain activation in response to seeing the protagonist on screen, seeing the antagonist on screen, auditory volume, visual complexity, and seeing violent acts. Shaded regions for control (*blue*) and Down syndrome (*red*) samples are thresholded at *p* < 0.001, FDR corrected. **b** Attention control network derived from functional connectivity in 1011 typically developing subjects to four seeds in the bilateral intraparietal sulcus and bilateral anterior insula with major hubs labeled

Activation maps for the five event-related contrasts studied (antagonist on screen, protagonist on screen, auditory, visual, and violence) are shown for Down syndrome and control samples in Fig. 1a, thresholded at *p* < 0.001, cluster corrected by false discovery rate, for display. Significant activation is more extensive for four of the contrasts in the control group than in the Down syndrome group but not for the protagonist on screen contrast (top row), in which the two samples show similar activation. Auditory and visual contrasts activate the primary auditory and primary and secondary visual cortex, respectively, with some activation of the visual cortex observed in controls for the auditory contrast as well. For protagonist on screen, antagonist on screen, and violence contrasts, the activated regions closely conformed to the expected location of the dorsal attention network, including bilateral intraparietal sulcus, frontal eye fields, and middle temporal cortex. This network is illustrated in Fig. 1b, derived from 1011 subjects from the 1000 Functional Connectomes dataset [14]. Activation of the dorsal attention network was spatially homologous between the two groups, and given that the primary contrasts associated with protagonist, antagonist, and violence recapitulated this network, with no significant activation of amydalar, orbitofrontal, insular or other emotive brain regions, the dorsal attention network was used as the primary metric in subsequent analyses.

**Fig. 2:**
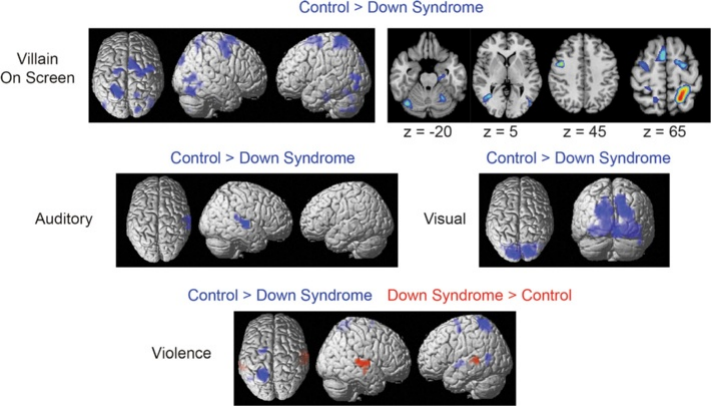
Differences in brain activation between Down syndrome and control samples. Brain regions showing greater activation for control participants are shown in rendered images (*upper left*) and slices (*upper right*) in response to seeing the antagonist on screen, with slice locations in radiological format given by MNI *z*-coordinate under the image. Brain regions showing significantly greater activation for control participants than for participants with Down syndrome are shown in the center in response to auditory volume and visual complexity of the video cartoons. Images showing greater activation for control individuals (*blue*) and individuals with Down syndrome (*red*) in response to violent scenes are shown in the image. All images are thresholded at *p* < 0.001, FDR corrected

To directly measure between-sample differences in activation, a two-tailed *t* test was performed for each of the five contrasts, and significant differences were observed for four of the contrasts, auditory, visual, violence, and antagonist on screen, with significant clusters reported in Table 2 and shown in Fig. 2 for each contrast. For the antagonist on screen contrast, control participants showed greater activation in the left entorhinal cortex as well as bilateral frontal eye fields, middle temporal, cerebellar hemisphere, and supplementary motor area. For the auditory contrast, control participants showed greater activation of the left primary auditory cortex. For the visual contrast, control subjects showed greater activation of most of the visual cortex, including striate and extrastriate cortex. For the violence contrast, control participants showed greater activation throughout a distributed set of brain regions closely aligned with the brain’s dorsal attention network. Participants with Down syndrome showed significantly greater activation of bilateral primary auditory cortex during violent scenes. None of the other contrasts showed any clusters with significantly higher activation in Down syndrome.

**Table 2:** Clusters showing significant difference in activation between control and Down syndrome groups. All clusters showed higher activation in control sample. *q* values show cluster-corrected false discovery rate. T-statistics show peak voxelwise results

Contrast	Region	MNI coordinates	Cluster *q*-value (FDR)	Number of voxels
Control > DS				
Antagonist on screen	Left intraparietal sulcus	(−21 − 55 67)	0.00000009	284
Antagonist on screen	Right frontal eye field	(18 –10 55)	0.000002	383
Antagonist on screen	Left frontal eye field	(−24 − 10 61)	0.008	80
Antagonist on screen	Left middle temporal	(−36 − 64 4)	0.000009	230
Antagonist on screen	Left parahippocampal	(−24 − 22 − 23)	0.011	66
Antagonist on screen	Left middle occipital	(−48 − 82 1)	0.016	57
Antagonist on screen	Left cerebellum	(−24 − 61 − 17)	0.008	76
Antagonist on screen	Right superior occipital	(18 − 91 34)	0.038	43
Antagonist on screen	Left cerebellum	(−12 − 73 − 47)	0.008	74
Antagonist on screen	Right supramarginal	(57 − 28 31)	0.06	34
Antagonist on screen	Right intraparietal sulcus	(18 − 61 61)	0.045	39
Auditory	Left superior temporal	(63 − 16 4)	0.001	117
Visual	Bilateral occipital	(18 − 76 37)	3.7 × 10^−28^	2428
Violence	Left intraparietal sulcus	(−18 − 55 73)	0.000008	229
Violence	Left posterior insula	(−36 − 13 1)	0.04	44
Violence	Left supramarginal	(−54 − 31 31)	0.05	33
Violence	Left frontal eye field	(−27 − 7 55)	0.04	43
Violence	Left middle temporal	(−39 − 58 1)	0.04	40
DS > control				
Violence	Right superior temporal	(63 − 19 4)	0.0001	153
Violence	Left superior temporal	(−57 − 40 4)	0.01	54

**Fig. 3:**
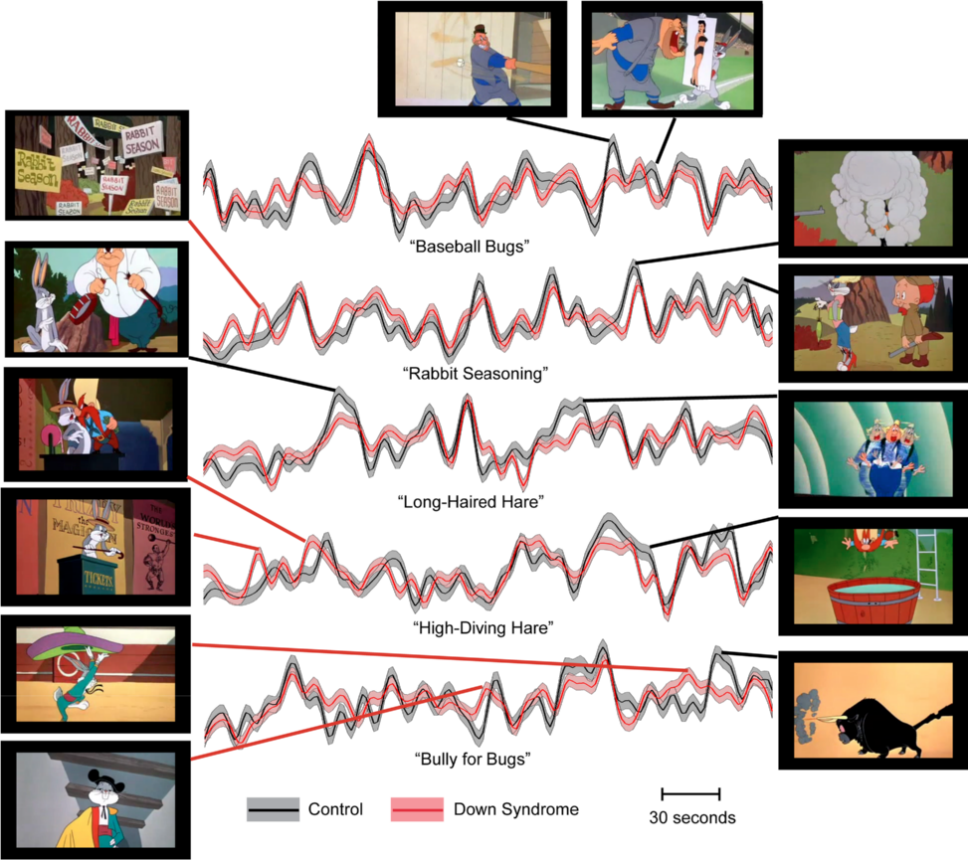
Mean activation of the dorsal attention network during five cartoons. *Shaded regions* show 95 % confidence intervals for the mean across participants in each sample. For scenes where the largest differences were seen between groups, representative screenshots are displayed from timepoints 6 s prior to peak activation (to account for hemodynamic response lag)

**Fig. 4:**
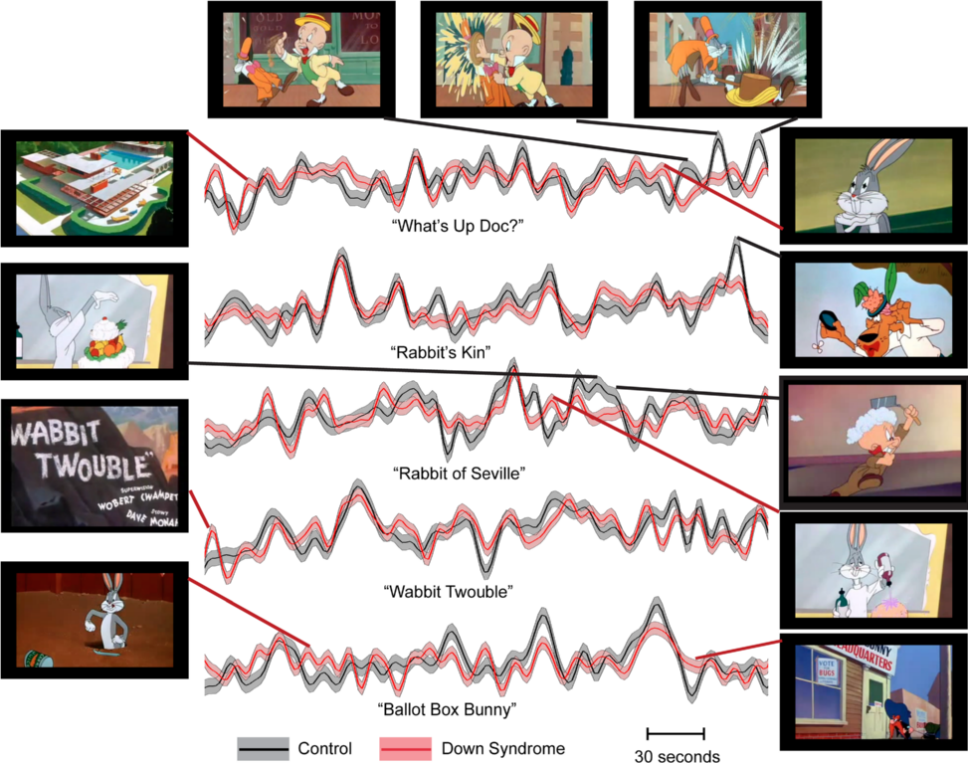
Mean activation of the dorsal attention network during five additional cartoons. *Shaded regions* show 95 % confidence intervals for the mean across participants in each sample. For scenes where the largest differences were seen between groups, representative screenshots are displayed from timepoints 6 s prior to peak activation (to account for hemodynamic response lag)

We next attempted to determine whether specific features of the cartoons resulted in the apparent decreased attentional network activation in Down syndrome. By creating a restriction mask from voxels in the dorsal attention network from the literature, we obtained average time series from this network for each participant, with mean time courses shown in Figs. 3 and 4 for Down syndrome and control samples. For timepoints where the 95 % confidence intervals were not overlapping and which showed the greatest differences between groups in relative dorsal attention network activation, screenshots display the content of the cartoons. Six seconds was subtracted from the timepoint in each case to correct for the effects of the hemodynamic response function. For all timepoints where 95 % confidence intervals did not overlap, a brief description of the scene is tabulated in Additional file 1: Tables S1 and S2.

Timepoints for which the control sample showed higher activation frequently correspond to activation peaks and occur at among the most violent scenes in the cartoons. In contrast, timepoints where participants with Down syndrome had higher activation are most typically at points where control participants show low activation, such as during opening credits (7 of 10 cartoons significantly higher in Down syndrome), or during scenes devoid of violence, such as Bugs Bunny dancing, closeup facial images of protagonists, or outdoor scenes. Of scenes where 95 % confidence intervals for mean dorsal attention network signal did not overlap between the two groups, 20 out of 37 of the scenes where control participants had higher signal involved violence, while only 1 out of 36 of the scenes where participants with Down syndrome had higher signal involved violence.

Only two cases of romantic or sexually themed content were present in the 10 cartoons showing differences in attentional activation between the groups: Bugs Bunny holding a pinup image to distract the catcher in a baseball game and Bugs Bunny dressing as a female to lure Elmer Fudd. In both cases, control participants showed significantly higher activation.

**Fig. 5:**
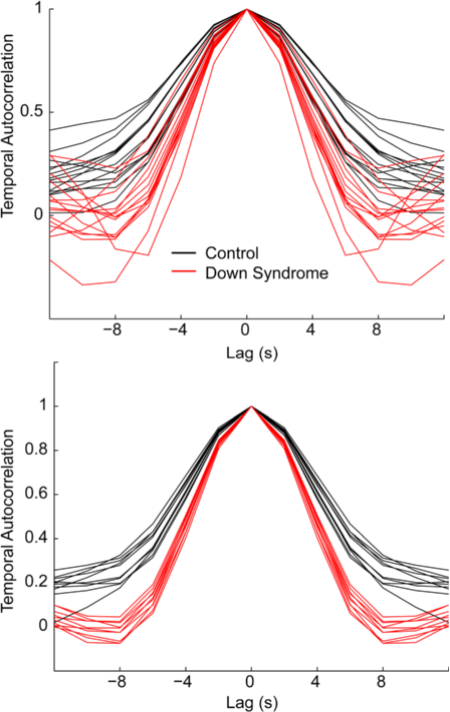
Temporal autocorrelation of the BOLD signal in the dorsal attention network. The autocorrelation is shown for each control and each participant with Down syndrome (*above*), averaged across all 10 cartoons. Each trace represents one participant. The mean autocorrelation across Down syndrome and control participants for each of the 10 cartoons is shown in the image, where each trace represents one cartoon

Comparisons of temporal autocorrelation during the cartoons are shown in Fig. 5. For all 10 cartoons, there was markedly narrower autocorrelation for participants with Down syndrome than for control participants. When evaluating individual participants, the full-width half maximum of autocorrelograms was significantly greater for the control group (mean control 2.60 ± 0.41 s.d., Down Syndrome 1.93 ± 0.26 s.d., *p* = 0.000016). This finding would be consistent with more idiosyncratic activation of the dorsal attention network or shorter periods of sustained activation within the network.

**Fig. 6:**
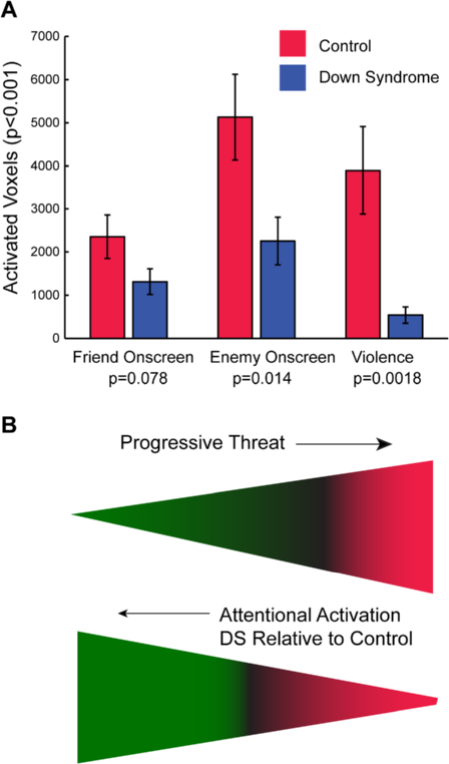
Brain activation in Down syndrome is specifically weaker as stimuli are more threatening. **a** Bar graph shows number of activated voxels for *p* < 0.001, uncorrected threshold, with *error bars* showing standard error of the mean across subjects. *P* values for two-tailed *t* test between groups are listed for each contrast below the labels. **b** As relative threat increases from seeing the protagonist to seeing the antagonist to seeing actual violent acts, the relative brain activation in individuals with Down syndrome compared to controls becomes smaller. For all three tasks, activated clusters are predominantly located in association cortex attentional regions

When activated voxels are tallied for control and individuals with Down syndrome, a progression is seen with increasing disparity between the two groups as the relative threat of the stimuli increases from seeing Bugs Bunny to seeing the antagonist to actual violence within the scene. This is illustrated quantitatively and graphically in Fig. 6.

## Discussion

In a sample of 15 Down syndrome and 14 typically developing control participants, we found that brain responses to passive cartoon viewing were largely similar between the groups, with similar brain regions that were activated despite marked functional impairment of the Down syndrome group. Nevertheless, brain activation to cartoon stimuli was significantly weaker in brain attentional regions in response to primary auditory and visual sensory stimulation, scenes involving the villain, and violent scenes in Down syndrome. The decreased responses to violence were specific to the extent that violent scenes could be predicted by differences in activation from moment to moment in the brain’s dorsal attention network. Brain responses to an “enemy” or “antagonist” present on screen were particularly low in Down syndrome in the dorsal attention network and left medial temporal lobe. These data provide some of the first evidence regarding the brain networks that may correspond to altered cognition in certain social cognitive situations in those with Down syndrome, such as those including negative emotions or violence. Dorsal attention network activation showed reduced temporal autocorrelation in Down syndrome, possibly consistent with shortened attention span or inability to sustain attentional activation over extended periods of time.

The brain’s dorsal attention network has been the subject of recent study in numerous reports. These regions, consisting of the bilateral intraparietal sulcus, frontal eye fields, and middle temporal (MT) regions, have been strongly implicated as the locus for attention to external stimuli [19] and show activation when an individual attends to audiovisual stimuli [15, 20]. This network is organized internally by sensory modality [21] and has shown a mutually anticorrelated relationship with the brain’s internal stimulus processing network, the default mode network [22, 23]. It is important to note that the brain regions of the dorsal attention network are polyfunctional and involve complex polymodal association cortex that can be implicated in other neural systems. Nevertheless, these regions are likely to provide a temporal marker for a high-attention state to external audiovisual stimuli.

It is striking that violent scenes predominantly activate this dorsal attention network. Although subjective experiences of the participants in the study cannot be directly inferred from our results, there is provocative evidence that the participants are paying unusual attention to violent scenes. Most of the relative peaks in the traces of Figs. 3 and 4 correspond to violent scenes, and the overall activation map is virtually overlaid with the anatomical boundaries of the dorsal attention network. The strong attentional focus on violent scenes among control individuals likely accentuates differences in attentional response in Down syndrome that may not be specific to violence, but in the context of these cartoon stimuli, it illustrate how generalized inattention in ecological social contexts may be most salient in relation to such emotively powerful events.

Prior reports of brain activation in response to media violence implicate several brain regions that may be more active during violent media viewing or associated with recent or chronic exposure to violent media. In one report of eight children, brain activation during viewing of a boxing scene from Rocky IV compared to viewing a nonviolent clip was greater in the right versus left precuneus, hippocampus, amygdala, and premotor cortex [24]. Another study evaluating responses to violence designed to evaluate desensitization overtime found activation to aggressive media scenes in bilateral orbitofrontal cortex, inferior frontal gyrus, anterior and posterior cingulated cortex, bilateral middle temporal, and bilateral middle occipital gyri [25].

In individuals with high exposure to media violence [26] and recent participation in violent video games, [27] activation to a Stroop task was differentially weaker in frontal lobe regions. Avid game players of violent video games exhibited decreased left lateral orbitofrontal activity in response to unpleasant visual stimuli compared to controls in one study [28]. Similarly, individuals with chronic high exposure to media violence exhibited weaker activation to violent images in the right lateral orbitofrontal cortex in another study [29]. In a placebo-controlled study in which participants played a violent video game after administration of quetiapine, an atypical antipsychotic agent, functional connectivity was decreased between the amygdala and the orbitofrontal cortex, with increased connectivity between amygdala and anterior cingulate and dorsolateral prefrontal cortex [30]. In this study, event-related BOLD signal for violent events was greater in the medial intraparietal sulci (visual attentional regions) [30]. A study analyzing PET responses to media violence in individuals with aggressive traits demonstrated decreased glucose metabolism in the medial orbitofrontal cortex for both violent and neutral stimuli [31]. Nevertheless, the brain imaging literature is virtually silent with respect to differences in brain responses to media violence associated with neurodevelopmental conditions. No literature was identified specifically evaluating how the brain is activated by stylized violence such as what is found in violent cartoons.

Medial temporal abnormalities in response to threat in Down syndrome are consistent with neuropsychological testing showing particular dysfunction associated with metrics requiring hippocampal and parahippocampal function [32]. This finding also reinforces approaches to investigation of treatments targeting medial temporal lobe function and supports findings of medial temporal dysfunction in a mouse model of Down syndrome [33]. We note that the medial temporal lobes process many functions that may contribute towards complex emotive responses including emotive salience, memory recall, theory of mind, and language. If the medial temporal lobe is relatively less engaged in responses to threats in Down syndrome, this may have profound effects throughout development on perception of risks of aversive situations, as well as the development of typical emotive responses to threat or impaired recall of semantic memories associated with a potential threat. Given that the brains of individuals with Down syndrome show particularly weak connectivity between distant brain regions, this may indicate a failure to integrate temporal lobe structures with distributed brain networks.

In addition, the failure to activate distributed brain attentional networks in response to threat may result from fundamental impairments in perception observed in visual and auditory cortex. Such primary perceptual deficits are not commonly observed in the literature for other neurodevelopmental disorders such as autism [34] and may represent a more profound functional brain disorder in Down syndrome consistent with the low IQ and more severe impairment of the individuals with Down syndrome in our study. Core perceptual abnormalities may be compounded for more complex attentional and cognitive processes that rely on perception. It seems likely that inattention may be a direct downstream consequence of perceptual activation and that the selective inattention to violence may be a consequence of the fact that violent scenes represent peak attentional events in control individuals. Additionally, attention to violence is a complex concept, integrating not only perception but language, memory, and theory of mind, all of which may be impaired in Down syndrome [35–37]. Other similarly complex social and behavioral interactions may exhibit similar accentuated inattention in Down syndrome, as evidenced by the poor attention to the two romantically themed episodes in our stimuli.

A failure of network-level integration may also be inferred from the reduced temporal autocorrelation in the dorsal attention network. Given that attentional network activation requires communication from disparate sets of loci throughout the brain, poor synchrony of these regions may result in an inability to sustain activations associated with conscious perception. Nevertheless, this possibility will require confirmation with more direct correlates of conscious perception, including subjective reports of experience or validation from other methods, since fMRI activation patterns do not unambiguously connote subjective experience and may be explained by other potential artifacts such as variable hemodynamic coupling or vascular differences. It is also possible that participants with Down syndrome systematically participated in the cartoon task differentially and that findings represent an epiphenomenon of participant involvement rather than abnormalities in brain network architecture. The participants with Down syndrome were relatively low functioning (many with IQ around 40, max full scale IQ 54), and the findings may also represent a nonspecific marker of low cognitive function.

It is intriguing that although decreased activation is seen for violent scenes and for scenes featuring the antagonist, participants with Down syndrome show no significant decreases in brain activation in response to Bugs Bunny. This may be consistent with the relatively pro-social features of Down syndrome personality spectra and reinforce the hypothesis that positive emotional stimuli may still engage attention networks despite intellectual dysfunction across multiple cognitive domains [38].

Additional studies may help to determine the range of experience over which individuals with Down syndrome may show medial temporal hypoactivation or decreased attention to threatening or violent stimuli. It is possible that our control cohort, with slightly higher IQ than the general population may limit external generalizability of our results. Because the dorsal attention network is defined in typically developing individuals, it is possible that subtle variation in the position or architecture of attentional processes in Down syndrome may underlie observed differences rather than behavioral responses. Nevertheless, Fig. 1 illustrates that the responses in the dorsal attention network appear spatially identical between groups, and similarly, the fact that cartoon viewers appear to show particular attention to violent scenes suggests that more evidence is required to understand the neurodevelopmental impact of violent media viewing. Nevertheless, it may be naïve in light of these results to assert that children or adults watching violent cartoons ignore or dismiss violent imagery.

## Conclusions

Typically developing adolescents and adults demonstrated strong, specific activation of the brain’s dorsal attention work in response to violent scenes in animated cartoons. In contrast, dorsal attention network responses in a sample of adolescents and young adults with Down syndrome showed significantly reduced activation in response to violent events during passive cartoon viewing compared to age-matched controls. Brain responses to the presence of an enemy or antagonist showed the greatest reduction within the dorsal attention network and left medial temporal lobe. Hypoactivation of both auditory and visual cortex was observed in response to cartoon viewing in Down syndrome. Finally, brain activation within the dorsal attention network showed reduced autocorrelation in Down syndrome, possibly representing a brain biomarker for reduced attention span in Down syndrome.

## Additional file

Additional file 1:
**Scenes where DS and control dorsal attention network activity differed.** **Table S1.** Cartoon scenes showing greater relative signal in the dorsal attention network for the Down syndrome sample than for the control sample. *P* value is calculated for two-tailed *t* test between subjects. Time is given in seconds from the onset of the cartoon. For scenes lasting longer than 2 s, the peak difference timepoint is shown in parentheses under Mean DS Signal. **Table S2.** Cartoon scenes showing greater relative signal in the dorsal attention network for the control sample than for the Down syndrome sample. *P* value is calculated for two-tailed *t* test between subjects. Time is given in seconds from the onset of the cartoon. For scenes lasting longer than 2 s, the peak difference timepoint is shown in parentheses under Mean DS Signal.
