# Supplementary material for: Violence: heightened brain attentional network response is selectively muted in Down syndrome
Source: J Neurodev Disord. 2015 Jun 3;7(1):15. doi: 10.1186/s11689-015-9112-y (PMC4486123; doi:10.1186/s11689-015-9112-y)
Supplement: Additional file 1: — Scenes where DS and control dorsal attention network activity differed. Table S1. Cartoon scenes showing greater relative signal in the dorsal attention network for the Down syndrome sample than for the control sample. P value is calculated for two-tailed t test between subjects. Time is given in seconds from the onset of the cartoon. For scenes lasting longer than 2 s, the peak difference timepoint is shown in parentheses under Mean DS Signal. Table S2. Cartoon scenes showing greater relative signal in the dorsal attention network for the control sample than for the Down syndrome sample. P value is calculated for two-tailed t test between subjects. Time is given in seconds from the onset of the cartoon. For scenes lasting longer than 2 s, the peak difference timepoint is shown in parentheses under Mean DS Signal. [file 11689_2015_9112_MOESM1_ESM.docx]

**Table S1.** Cartoon scenes showing greater relative signal in the dorsal attention network for the Down Syndrome sample than for the control sample. p-value is calculated for two-tailed t-test between subjects. Time is given in seconds from the onset of the cartoon. For scenes lasting longer than 2 seconds, the peak difference timepoint is shown in parentheses under Mean DS Signal.

| Scene Description | Cartoon | Time | Violent | p-value | Mean DS Signal | Mean HC Signal |
| --- | --- | --- | --- | --- | --- | --- |
| The “Gashouse Guerilla” tosses the baseball equipment onto Bugs Bunny and tells Bugs Bunny, “You’ve got yourself a game.” | “Baseball Bugs” | 142 | No | .048639 | .38396 | -.22836 |
| A sign appears that reads, “ IF YOURE LOOKING FOR FUN-,“ right after the opening credits end. | “Rabbit Seasoning” | 12-16 | No | .015633 | -.083604  (14) | -.92034 |
| The music becomes dramatic and a myriad of signs appear that all read, “Rabbit Season.” | “Rabbit Seasoning” | 30-32 | No | .010387 | .30861  (32) | -.55414 |
| Elmer Fudd tells the viewers that it’s rabbit season as he puts his head down to start looking for rabbit tracks. | “Rabbit Seasoning” | 74-76 | No | .0051424 | .45055  (76) | -.42681 |
| Bugs Bunny and Elmer Fudd lift their heads from the rabbit hole after Bugs Bunny says his signature phrase, “What’s up Doc?” | “Rabbit Seasoning” | 98 | No | .030069 | .26464 | -.41279 |
| Daffy Duck tells Bugs Bunny that he’s not going to fall for Bugs Bunny’s trickery again. | “Rabbit Seasoning” | 208 | No | .049071 | .11039 | -.49631 |
| Opening credits changing on the screen. | “Long-Haired Hare” | 10-14 | No | .0058162 | -.27708  (12) | -1.1434 |
| The maestro practices his singing and looks somewhat angry over the presence of Bugs Bunny. | “Long-Haired Hare” | 88 | No | .039896 | -.24124 | -.82603 |
| Bugs Bunny sings a charming song on the harp and seconds later the maestro mockingly dances to the song. | “Long-Haired Hare” | 100-104 | No | .0062884 | .25469  (102) | -.55824 |
| Bugs Bunny, disguised as a fan, pleads for Mr. Jones (the maestro) to not go on stage. | “Long-Haired Hare” | 240 | No | .034521 | .18002 | -.47219 |
| The music is playing loudly and the maestro angrily stares at the audience since he’s continually fallen for Bugs Bunny’s tricks. | “Long-Haired Hare” | 266 | No | .042507 | .34138 | -.35799 |
| Bugs Bunny originally appears eating a carrot, and then the title “HIGH DIVING HARE” appears. | “High-Diving Hare” | 0-2 | No | .017704 | -.00764  (0) | -.7873 |
| Bugs Bunny tells a large crowd about how everyone in the crowd should buy tickets to a spectacular event. | “High-Diving Hare” | 26-30 | No | .0081601 | .47285  (28) | -.57619 |
| Bugs Bunny tells the crowd that they can watch all “15 sensational acts for 15 cents,” which causes Yosemite Sam to jump out of his seat. | “High-Diving Hare” | 56-58 | No | .0073963 | .8428  (58) | -,066981 |
| Bugs Bunny emerges from the trail in the ground and starts complaining that he’s in the wrong location. | “Bully for Bugs” | 66-68 | No | .0096002 | -09159  (68) | -,93312 |
| Bugs Bunny takes out a map and laments that he made the wrong turn at Albuquerque. | “Bully for Bugs” | 74-76 | No | .015176 | -.0315  (76) | -.65463 |
| The bull runs full-speed at Bugs Bunny, but Bugs Bunny, dressed as a matador, looks unconcerned. | “Bully for Bugs” | 146-150 | No | .0017634 | .15796  (148) | -.8625 |
| Bugs Bunny mocks the bull and hides under a hat after performing a dance. Bugs Bunny then pinches his nose, and moments later the bull is seen sharpening his horns again. | “Bully for Bugs” | 254-264 | Yes | .0035454 | .65959  (256) | -.34492 |
| A picture of the title along with Bugs Bunny followed by opening credits. | “What’s Up Doc?” | 6-8 | No | .041524 | .28394  (8) | -.76413 |
| Music plays with scenery in the background followed by the camera moving in on a big house. | “What’s Up Doc?” | 22-24 | No | .0050691 | -.06806  (24) | -1.0093 |
| Bugs Bunny is retelling his life story and he’s shown playing the piano as a baby rabbit. | “What’s Up Doc?” | 76 | No | .029876 | .49991 | -.3442 |
| A green flashing sign that reads, “Wearing of the Grin.” | “What’s Up Doc?” | 130-132 | No | .0069181 | .40731  (132) | -.32795 |
| Bugs Bunny’s eyes turn into stars when Elmer Fudd tells him that he’s a star and that he’ll give him equal billing. | “What’s Up Doc?” | 246 | No | .030569 | .31104 | -.56376 |
| The camera focuses on Bugs Bunny as he curiously stares at the puma. | “Rabbit’s Kin” | 136 | No | .026254 | -.022116 | -.68816 |
| The puma, disguised as the little rabbit’s mother, tells Bugs Bunny that “she” would like her little rabbit back, although it is obvious that the disguise is fake. | “Rabbit’s Kin” | 214 | No | .036779 | .55013 | -.044532 |
| Bugs Bunny talks with the puma with the camera focused on Bugs Bunny. | “Rabbit’s Kin” | 260 | No | .02111 | .068291 | -.56401 |
| Elmer Fudd searches for Bugs Bunny in a building with a gun in his hand so he can shoot him. | “Rabbit of Seville” | 66 | No | .012519 | .46949 | -.24467 |
| Bugs Bunny is in the middle of his musical act and sees Elmer Fudd walk out onto the stage, so he sings, “Hey you,” and stops him from leaving so he can use him in the act. | “Rabbit of Seville” | 96-98 | No | .017537 | .75575  (96) | .06448 |
| Bugs Bunny gets a puzzled expression on his face when he tells Elmer Fudd that his face might have gone through a machine. | “Rabbit of Seville” | 132 | No | .038518 | -.30998 | -.90648 |
| Bugs Bunny disguises himself in a green outfit and sings to Elmer Fudd as a part of the musical. | “Rabbit of Seville” | 144 | No | .049277 | -.19201 | -.69402 |
| Bugs Bunny pours shampoos on Elmer Fudd’s head with a strange expression on his face. | “Rabbit of Seville” | 182-186 | No | .0022827 | .30372  (184) | -.62185 |
| Elmer Fudd tells himself about how lovely the campsite is as the camera shows the trees above him. | “WabbitTwouble” | 148-152 | No | .012639 | -.19011  (148) | -.92404 |
| Yosemite Sam talks loudly to potential voters about how they live in such a great country. | “Ballot Box Bunny” | 30 | No | .042555 | .35455 | -.38691 |
| Bugs Bunny sticks his hand up in the air and says that he’s going to fight Uncle Sam’s claim that the town is going to get rid of the rabbits. | “Ballot Box Bunny” | 54-56 | No | .017914 | .28077  (56) | -.62196 |
| Yosemite Sam screams about how much he hates Bugs Bunny after getting tricked again, and then the scene changes and Yosemite Sam is seen carrying a cannon. | “Ballot Box Bunny” | 258-260 | No | .025036 | -.080268  (260) | -.9451 |
| Bugs Bunny tells Yosemite Sam that the person knocking on the door was for him. | “Ballot Box Bunny” | 300 | No | .043025 | -.14065 | -.7188 |

**Table S2.** Cartoon scenes showing greater relative signal in the dorsal attention network for the control sample than for the Down Syndrome sample. p-value is calculated for two-tailed t-test between subjects. Time is given in seconds from the onset of the cartoon. For scenes lasting longer than 2 seconds, the peak difference timepoint is shown in parentheses under Mean DS Signal.

| Scene Description | Cartoon | Time | Violent | p-value | Mean HC Signal | Mean DS Signal |
| --- | --- | --- | --- | --- | --- | --- |
| Bugs Bunny throws a baseball in slow motion and all of the “Gashouse Guerillas” swing and miss the ball, which frustrates them to the extent that the last batter throws down his bat in rage. | “Baseball Bugs” | 216-220 | No | .0029952 | 1.2728  (218) | .42564 |
| Bugs Bunny gets into the mask of the umpire (a “Gashouse Guerilla” in disguise) to argue that he was safe at the plate. | “Baseball Bugs” | 266 | No | .030506 | .057551 | -.76985 |
| Elmer Fudd shoots Daffy Duck in the face. | “Rabbit Seasoning” | 146 | Yes | .025671 | 1.1171 | .44982 |
| Elmer Fudd shoots Daffy Duck for the third and final time after Daffy Duck asks him what he would do if he (Daffy Duck) were a rabbit. | “Rabbit Seasoning” | 222-228 | Yes | .00019408 | 1.2647  (226) | .051433 |
| Elmer Fudd shoots at Bugs Bunny and Daffy Duck as they run for their lives. | “Rabbit Seasoning” | 246 | Yes | .03049 | .532 | -.0736 |
| Daffy Duck lifts his head up out of the rabbit hole to see if Elmer Fudd is still outside, and a gunshot goes off and it is obvious that Daffy Duck has been shot yet again. | “Rabbit Seasoning” | 260-262 | Yes | .031222 | 1.1524  (262) | .51122 |
| Bugs Bunny comes out of the rabbit hole disguised as a woman, and Elmer Fudd thinks that “she” is beautiful and catcalls to Bugs Bunny. | “Rabbit Seasoning” | 286-288 | No | .0072633 | 1.0096  (286) | .09398 |
| Daffy Duck yells at Elmer Fudd as he tries to tell him that Bugs Bunny’s disguise shouldn’t trick him. | “Rabbit Seasoning” | 298-300 | No | .028424 | .19997  (300) | -.60826 |
| The maestro practices for his recital by working on his voice while Bugs Bunny can be heard lightly singing in the background. | “Long-Haired Hare” | 38 | No | .023502 | -.34219 | -1.1956 |
| The maestro takes Bugs Bunny ukulele and violently shatters it to pieces. | “Long-Haired Hare” | 70-76 | Yes | .016913 | 1.3252  (70) | .57647 |
| The maestro begins singing at his concert. | “Long-Haired Hare” | 170-172 | No | .0093665 | -.44914  (170) | -1.2918 |
| The maestro bounces around the stage after Bugs Bunny slams it with a hammer, causing it to vibrate. The maestro ends up screaming for help in a trombone. | “Long-Haired Hare” | 188-198 | Yes | .00035067 | 1.0744  (192) | -.08885 |
| An explosion occurs after Bugs Bunny, disguised as a fan, asks the maestro for an autograph, but uses dynamite instead of a pen. | “Long-Haired Hare” | 258 | Yes | .028255 | .84951 | .16012 |
| Bugs Bunny explains to the crowd that the Fearless Freep will dive off the 500-foot high dive. | “High-Diving Hare” | 102-106 | No | .0012872 | .24593  (104) | -.61958 |
| Bugs Bunny learns that Fearless Freep can’t perform and gulps, as he knows that he’s going to upset the crowd. | “High-Diving Hare” | 118 | No | .04102 | -.17993 | -.85718 |
| Bugs Bunny pretends to jump off the high dive for Yosemite Sam but everyone (including the viewer) can see that he is safe and just making sound effects. | “High-Diving Hare” | 214-220 | No | .012687 | 1.4069  (216) | .75243 |
| Yosemite Sam freefalls from the high dive after getting tricked by Bugs Bunny and shatters the water tank at the end of the fall. | “High-Diving Hare” | 232-236 | Yes | .0064069 | .52871  (234) | -.44182 |
| Yosemite Sam freefalls from the high dive after getting tricked by Bugs Bunny again as Bugs Bunny tries to pour water into the bucket before Yosemite Sam lands in an empty bucket. | “High-Diving Hare” | 268-270 | Yes | .022606 | 1.0964  (270) | .31852 |
| Yosemite Sam misses the tank and crashes into the floor after missing the water bucket. | “High-Diving Hare” | 280-282 | Yes | .022507 | 1.0962  (280) | .18313 |
| The bull knocks Bugs Bunny unconscious after hitting him on the head. | “Bully for Bugs” | 200 | Yes | .027397 | .97181 | .45248 |
| The bull slams Bugs Bunny into the wall, which makes Bugs Bunny see stars before passing out. | “Bully for Bugs” | 224-226 | Yes | .018292 | -.0076854  (226) | -.65668 |
| The bull runs through Bugs Bunny booby-trap and ends up with a gun up his rear-end, which fires off a bullet that goes through the bull’s horn. | “Bully for Bugs” | 272-278 | No | .0019735 | 1.2048  (274) | .090804 |
| Elmer Fudd hits Bugs Bunny in the face and then sprays him with water after Bugs Bunny intentionally asked a stupid question as a part of the act. | “What’s Up Doc?” | 254-260 | Yes | .0055848 | .3785  (256) | -.69559 |
| Elmer Fudd slams Bugs Bunny with a pie in the face and laughs at him as a part of the act. | “What’s Up Doc?” | 272-276 | Yes | .0066825 | 1.2297  (274) | .047182 |
| Bugs Bunny turns the tables on Elmer Fudd by slamming a pie in his face, spraying him with water, and bashing him with a hammer. | “What’s Up Doc?” | 292-294 | Yes | .022699 | 1.1451  (294) | -.1165 |
| Bugs Bunny throws out his hands and tells the puma that the puma is free to eat him, although it is obvious that Bugs Bunny isn’t worried. | “Rabbit’s Kin” | 124-126 | No | .0062312 | .32245  (124) | -.42516 |
| Bugs Bunny uses an “ACME STOVELID LIFTER” to make the lumps on the puma’s head emerge after Bugs Bunny hit the puma with a hammer. | “Rabbit’s Kin” | 282-286 | Yes | .0080979 | 1.7351  (284) | .62551 |
| Elmer Fudd fires his gun but it backfires and hits him instead since he didn’t realize Bugs Bunny made the gun into a knot. | “Rabbit of Seville” | 176-178 | Yes | .0025935 | .66517  (176) | -.20477 |
| Bugs Bunny makes a fruit salad on top of the unconscious Elmer Fudd and tops off the salad with some whip cream. | “Rabbit of Seville” | 202-214 | No | .0019867 | .88172  (210) | -.07455 |
| Elmer Fudd chases Bugs Bunny with a butcher’s knife. | “Rabbit of Seville” | 218 | Yes | .037321 | .57628 | .045721 |
| Bugs Bunny runs in terror after Elmer Fudd shoots down the shocker and leaves Bugs Bunny defenseless. | “Rabbit of Seville” | 240 | Yes | .01244 | .3025 | -.39008 |
| Elmer Fudd says to himself that he has to go to bed because he thinks its nighttime, even though it’s still daytime. | “WabbitTwouble” | 182-188 | No | .026305 | .87334  (186) | .23505 |
| Elmer Fudd washes his face in the sink and searches for his towel so he can dry off his face. | “WabbitTwouble” | 244-250 | No | .038281 | .32383  (246) | -.4251 |
| Elmer Fudd chases Bugs Bunny after Bugs Bunny admits that he tricked him. | “WabbitTwouble” | 288-292 | No | .0067237 | .194  (290) | -.68109 |
| Bugs Bunny, dressed as Theodore Roosevelt, uses Roosevelt’s signature phrase: “I speak softly, but carry a big stick.” | “Ballot Box Bunny” | 102 | No | .047848 | .01385 | -.7535 |
| Bugs Bunny, disguised as a baby, gives Yosemite Sam a big kiss after Yosemite Sam said that he loved babies to help him win the election. | “Ballot Box Bunny” | 132-136 | No | .04011 | .87757  (132) | .28827 |
| Yosemite Sam helps light a cigar for a potential voter, and the cigarette explodes when it reaches the end. The viewers already know that this incident was destined to happen as Bugs Bunny replaced the regular cigars with exploding cigars. | “Ballot Box Bunny” | 178-186 | Yes | .0041274 | 1.3016  (182) | .52005 |
